# Associations Between Sexual Orientation and Self-Perceptions of Aging: The Withering Role of Stress

**DOI:** 10.1093/geroni/igaf122.934

**Published:** 2025-12-31

**Authors:** Anyah Prasad, Serena Sabatini

**Affiliations:** Vanderbilt University, Boston, Massachusetts, United States; University of Barcelona, Barcelona, Catalonia, Spain

## Abstract

Self-perceptions of Aging (SPAs) are an overarching term encompassing different aspects of people’s awareness of aging, including their own internal aging processes, changes in their social roles/relationships, and the broader cultural notions of aging. Generally, people with more positive SPAs tend to be healthier and live longer. This study uses nationally representative data from the German Ageing Survey (wave 2021) to investigate the differences between sexual minority and heterosexual respondents on multiple dimensions of SPAs and examine the mediating role of stress. Two hundred and fifty seven sexual minority respondents were matched one-to-one with heterosexual respondents based on their age (mean 72 years) and sex (45% women). Cross-sectional linear regression and mediation models were estimated. Sexual minority respondents had more negative attitudes towards own aging (Standardized β=-0.14, p = 0.001) and reported more perceived social losses (β = 0.11, p = 0.017) than heterosexual respondents. Sexual orientation was not significantly associated with felt age, perceived physical losses, personal growth, and self-knowledge. Stress fully mediated the cross-sectional association of sexual orientation with attitudes towards own aging, and perceived social losses. Although causality cannot be inferred, higher stress may explain why sexual minority respondents may experience more negative attitudes towards own aging and social losses. SPAs can be used as a screening tool to identify sexual minority older adults at most risk. Interventions to reduce stress and improve SPAs may help in supporting sexual minority individuals’ future wellbeing.

